# Understanding the Nature of Hybrid Sterility and Divergence of Asian Cultivated Rice

**DOI:** 10.3389/fpls.2022.908342

**Published:** 2022-06-27

**Authors:** Yu Zhang, Jie Wang, Qiuhong Pu, Ying Yang, Yonggang Lv, Jiawu Zhou, Jing Li, Xianneng Deng, Min Wang, Dayun Tao

**Affiliations:** ^1^Yunnan Key Laboratory for Rice Genetic Improvement, Food Crops Research Institute, Yunnan Academy of Agricultural Sciences (YAAS), Kunming, China; ^2^Institute of Plant Resources, Yunnan University, Kunming, China

**Keywords:** *Oryza sativa* L., hybrid sterility, subgroup, divergence, heterosis

## Abstract

Intraspecific hybrid sterility is a common form of postzygotic reproductive isolation in Asian cultivated rice, which is also the major obstacle to utilize the strong heterosis in the rice breeding program. Here, we review recent progress in classification and hybrid sterility in Asian cultivated rice. A genome-wide analysis of numerous wild relatives of rice and Asian cultivated rice has provided insights into the origin and differentiation of Asian cultivated rice, and divided Asian cultivated rice into five subgroups. More than 40 conserved and specific loci were identified to be responsible for the hybrid sterility between subgroup crosses by genetic mapping, which also contributed to the divergence of Asian cultivated rice. Most of the studies are focused on the sterile barriers between *indica* and *japonica* crosses, ignoring hybrid sterility among other subgroups, leading to neither a systematical understanding of the nature of hybrid sterility and subgroup divergence, nor effectively utilizing strong heterosis between the subgroups in Asian cultivated rice. Future studies will aim at identifying and characterizing genes for hybrid sterility and segregation distortion, comparing and understanding the molecular mechanism of hybrid sterility, and drawing a blueprint for intraspecific hybrid sterility loci derived from cross combinations among the five subgroups. These studies would provide scientific and accurate guidelines to overcome the intraspecific hybrid sterility according to the parent subgroup type identification, allowing the utilization of heterosis among subgroups, also helping us unlock the mysterious relationship between hybrid sterility and Asian cultivated rice divergence.

## Introduction

Biological species form when the gene flow between two divergent populations has mostly or completely limited, and the striking characteristic of species is that species can actually or potentially interbreed, but are prevented from producing the fertile progeny with other species (Paterson, 1985). Reproductive isolation is critical for the formation and maintenance of species between different populations (Oka, 1988). It could occur at different developmental stages, including prezygotic and postzygotic reproductive isolation, the former prevents mating or formation of hybrid and the latter reduces the fitness of hybrid (Seehausen et al., 2014). Hybrid sterility is a major form of postzygotic reproductive isolation in rice and has been a good example of genetic studies of population speciation as an intrinsic genetic factor (McDermott and Noor, 2010; Ouyang et al., 2010; Ouyang and Zhang, 2013).

Genus *Oryza* contains 23 wild species and two cultivated rice species with 11 genome types. Of the 11 genome types, 6 are diploid, including AA, BB, CC, EE, FF, and GG, and five are polyploidy, including BBCC, CCDD, HHJJ, HHKK, and KKLL (Khush, 1997; Stein et al., 2018). Six wild species of *Oryza nivara, Oryza rufipogon, Oryza barthii, Oryza glumaepatula, Oryza longistaminata*, and *Oryza meridionalis*, as well as the two cultivated species of *Oryza sativa* and *Oryza glaberrima* were classified into the AA genome (Khush, 1997). Thus, AA genome species were considered as the natural germplasm pool for rice genetic improvement (Vaughan, 1994). However, hybrid sterility occurs widely in intraspecific and interspecific hybrids in rice, which is the major barrier to utilize heterosis and make genetic improvement difficult by restricting gene exchange in the Asian cultivated rice, African cultivated rice, and between the two cultivated species and the wild relatives (Ouyang and Zhang, 2013; Li et al., 2020).

This review summarized recent progress in understanding the genetic differentiation in Asian cultivated rice, expatiated on the genetic basis of hybrid sterility between different subpopulations, discussed the role of hybrid sterility on the population divergence, as well as tried to outline the future efforts to be done in intraspecific hybrid sterility study.

## Genetic Differentiation of the Asian Cultivated Rice

Asian cultivated rice (*O. sativa* L.) is widely cultivated around the world and mainly in Asia. Abundant genetic diversity and significant genetic differentiation due to the different geographic or ecological adaptation, as well as domestication, had been reported (Huang et al., 2011). The two distinguishable groups were recognized as “*hsien*” and “*Keng*” in Chinese ancient records from the Han dynasty (Ting, 1949a,b,c). These two different groups of *O. sativa* subs. *japonica* (*Keng/Geng*) and *indica* (*hsien/Xian*) were first named based on their morphological and serological characteristics, together with hybrid fertility differences (Kato et al., 1928), consistent with the classification by the ancient Chinese people. Then, distinct classification results emerged in view of different criteria. The three types, A, B, and C, were used to classify *O. sativa* according to the different geographical distribution, later regarded as *japonica, javanica*, and *indica* (Matsuo, 1952; Morinaga, 1954). Further studies indicated that Asian cultivated rice should be classified into three types and five subgroups according to hybrid affinity ability and geographic distributions. Japanese rice belonged to the *japonica* type, aman and tjereh subgroup belonged to *indica* type because of the low affinity of the Japanese subgroup, whereas aus together with bulu subgroup could be reconsidered as neither *japonica* nor *indica*, because they showed considerable high hybrid fertility to both types, so they were called the intermediate type. Thus, *O. sativa* might be grouped into three types: *japonica, indica*, and intermediate types (Morinaga and Kuriyama, 1955, 1958). A fast and feasible criterion for distinguishing *Keng* (*japonica*) from *hsien* (*indica*) based on some classification traits was established, such as apiculus hair length, grain size, hull color at heading, phenol reaction, and other traits, some uncertain intermediate varieties were assessed followed by traits of leaf hair and 1–2 internode length of the main panicle. This method, called the “Cheng's index,” was effective in practice (Cheng et al., 1984). Isozyme was also used to study the differentiation of *O. sativa* at the biochemical level. One thousand six hundred and eighty-eight Asian varieties were detected by 15 isozyme markers at eight loci, the result indicated that Asian cultivated rice was divided into six different groups: I, II, III, IV, V, and VI, respectively, corresponding *indica, aus, deepwater* rice in Bangladesh, *deepwater* rice in Northeast India, *basmati*, and *japonica* (Glaszmann, 1987). Reviewing the previous results, nearly all the researchers agreed with the opinion that Asian cultivated rice contained two major groups: *japonica* and *indica*, but the classification of intermediate groups was ambiguous. The limited number or geographic distribution of varieties, evaluated by a few traits and genetic markers, ignoring the role of origin, domestication, and selection on the divergence of *O. sativa*, resulted in the un-uniformed classification of *O. sativa*.

Progress in the development of a great number of molecular markers and genome sequencing provided a new insight into understanding the differentiation of *O. sativa* at the molecular level. Two hundred and thirty-four rice varieties representing a wide geographic range of *O. sativa* were genotyped using 169 nuclear SSRs and two chloroplast loci; the results indicated that five distinct groups were detected, corresponding to temperate *japonica*, tropical *japonica, indica, aus*, and *aromatic* rice (Garris et al., 2005). Twenty diverse varieties and landraces by genotyping 160,000 non-redundant SNPs not only fitted well with five groups of *O. sativa*, but also demonstrated genomic introgression from one varietal group into another under strong artificial selection for production or environment adaptation demands (McNally et al., 2009). The analysis of the genetic variation of 3,010 Asian cultivated rice accessions by genome-wide SNP supported the classification of the five major groups in *O. sativa*, but also revealed seven unreported and geographic distribution-related subgroups (Wang et al., 2018). Population genetics of 5,152 rice accessions analyzed by resequencing and genetic diversity analysis of 60 rice varieties using 190 SNPs had clearly demonstrated the genetic difference among temperate *japonica*, tropical *japonica, indica, aus*, and *aromatic* rice (Kishor et al., 2020; Yan et al., 2020). Taken together, the population structure and genetic diversity of the worldwide rice gave insights into the five distinct subgroups; this classification of *O. sativa* was widely recognized by rice scientists.

Basmati rice is the typical representative of the *aromatic* type, and the fragrance trait can be found in *basmati* (Dom-sufid from Iran), *indica* (KDM105 from Thailand), and tropical *japonica* (Azucena from Philippines) (Bradbury et al., 2005; Bourgis et al., 2008; Kovach et al., 2009; Myint et al., 2012); therefore, the word “*aromatic*” representing one of the five subgroups is easily confused. Here, we use *basmati* to be the *japonica-*clinous rice from Myanmar to South Asia and Iran so as to discriminate *aromatic* rice from *indica*, tropical *japonica*, temperate *japonica*, and *aus*.

**Figure 1:**
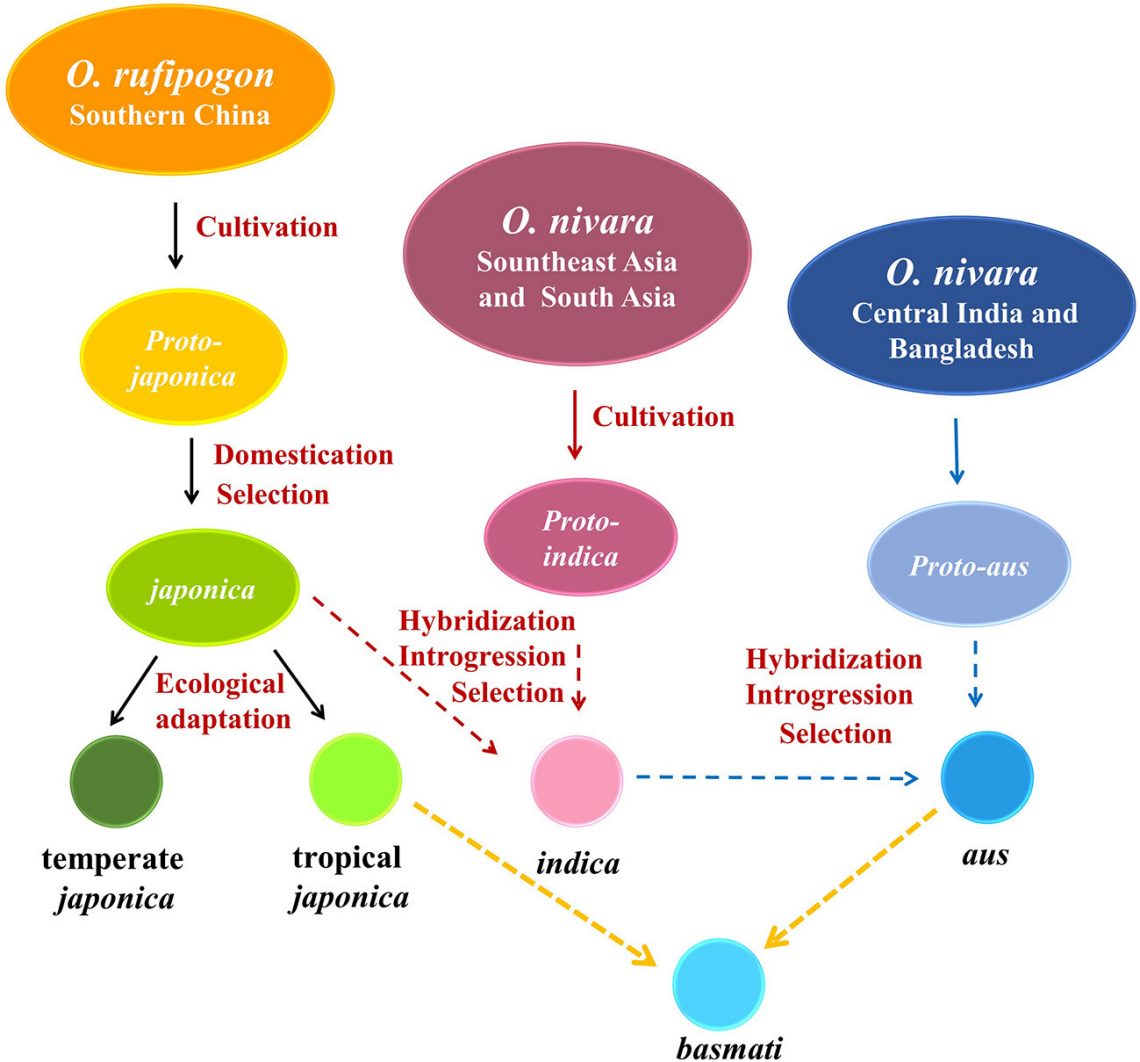
Origin and domestication model of Asian cultivated rice. Archaeological and genetic evidence indicated that *japonica* rice originated from a *O. rufipogon* population around the middle area of the Pear River and began to be cultivated about 9,000 years BP in the Yangtze Valley in southern China; *japonica* spread to South Asia about 4,000 years BP leading to the introgression of domestication genes into *pro-indica* derived from local *O. nivara* in Southeast Asia and South Asia, and *indica* was then formed to be cultivated in the lower Ganges valley (Zhao, 2011; Huang et al., 2012; Gross and Zhao, 2014; Stein et al., 2018). Tropical *japonica* and temperate *japonica* differed genetically due to a global reduction in temperature and humidity; it was the result of strong pressure to adapt to different climatic conditions (Kovach et al., 2007; Gutaker et al., 2020). Recently, two additional subspecies of Asian cultivated rice, *aus* and *basmati*, were recognized to differentiate distinctly from *japonica* and *indica*. *O. nivara* in central India and Bangladesh as the gene pool crossed with *indica* and selected to adapt to rainfed conditions, including rainfed upland, rainfed lowland, and deep-water, gave rise to *aus* group, whereas *aromatic* was the hybrid of *aus* and tropical *japonica* and was popular in Pakistan, northern India (*basmati*), and Iran (sadri) (Khush et al., 2013; Civan et al., 2015; Travis et al., 2015; Choi et al., 2020).

In addition, a high-throughput genome sequence, coupled with archaeobotanical, paleoclimate, and geographic data, revealed the origin and domestication history of the five subgroups of *O. sativa*, thus supporting multiple origins and at least one domestication in Asian cultivated rice, which was accepted by scientists (Zhao, 2011; Huang et al., 2012; Gross and Zhao, 2014; Civan et al., 2015; Travis et al., 2015; Stein et al., 2018; Choi et al., 2020; Gutaker et al., 2020; Figure 1), but this classification was not proved by reproductive isolation.

## Genetic and Molecular Basis of Hybrid Sterility in Asian Cultivated Rice

### Main Features of Hybrid Sterility in Asian Cultivated Rice

Hybrid sterility is widely observed in rice, causing reduced gamete viability of hybrids between divergent subspecies. Hybrid sterility between *japonica* and *indica* was first reported when Asian cultivated rice was classified into the two subspecies using the criterion of hybrid fertility (Kato et al., 1928). Since then, the genetic basis of hybrid sterility had been investigated and scientists found some distinguished features in hybrid sterility: (1) Cytoplasm had almost no effect on hybrid fertility because reciprocal crosses showed no significant difference in male and female gametes fertility of F_1_ plants (Kato, 1930; Oka, 1957); (2) F_1_ hybrids between *indica* and *japonica* were usually sterile, the hybrid fertility varied dependent on the number of hybrid sterility genes and genetic distance between the two parents (Oka, 1988; Zhang et al., 1989, 1994); (3) Sterility could be explained by hybrid sterility genes instead of chromosome disturbance (Kato, 1930); (4) The F_1_ and F_2_ fertilities are not correlated (Oka and Doida, 1962); (5) Hybrid sterility between *japonica* and *indica* could be observed due to male gamete abortion, female gamete abortion, and embryo abortion (Teng et al., 1996; Zhu et al., 1996; Liu et al., 2004); (6) Hybrid gene interactions, including both allelic interaction at the same locus and non-allelic interactions at the different loci, were responsible for varying degrees of hybrid sterility in crosses between subgroups of *O. sativa* (Zhang and Lu, 1993; Zhang et al., 1993; Kubo et al., 2008).

### Identification, Cloning, and Molecular Characterization of Hybrid Sterility Loci

By now, more than 40 loci responsible for hybrid sterility in the crosses between the different subgroups are identified by genetic mapping in rice (Supplementary Table 1). Surprisingly, three loci, *Ef1, Pf3*, and *Sf3*, were found in the cross between ZS97 and Minghui 63, which were famous *indica* parents in hybrid rice breeding. It is suggested that hybrid sterility also happened within the *indica* cross (Supplementary Table 1). But it was notable that ZS97 and Minghui 63 were not traditional varieties, they were the product of modern breeding incorporating other genome introgression fragments from other subgroups by multiple round crosses (McNally et al., 2009); therefore, cloning and gene sequence analysis of three loci will answer whether those loci were responsible for hybrid sterility in *indica* × *indica* cross or not. Moreover, there is no gene or QTL identified in the cross between *basmati* and other subgroups, further study will pay more attention to identifying, cloning, and characterizing the molecular mechanism of hybrid sterile genes from *basmati* rice.

Until now, six loci for intraspecific hybrid sterility were cloned and characterized. *Sa* was the first characterized hybrid male sterility loci between *indica* and *japonica*. SaM encoded a small ubiquitin-like modifier E3 ligase, and SaF encoded an F-box protein. Three divergent genetic components: *SaF*^+^ (the *indica* allele), *SaM*^+^ (the *indica* allele), and *SaM*^−^ (the *japonica* allele) consisted of a killer system to selectively abort the male gametes harboring *SaM*^−^ in the *indica*×*japonica* hybrid (Long et al., 2008). *S5* locus conferring female hybrid sterility consisted of three tightly linked genes, *ORF3, ORF4*, and *ORF5*, which acted in a killer–protector system. ORF3, ORF4, and ORF5 encoded an HSP70 heat shock protein, a transmembrane protein, and a putative aspartic protease, respectively. During female development, endoplasmic reticulum (ER) stress is caused by the action of *ORF5*+ (killer) and *ORF4*+ (partner), and *ORF3*+ (protector) could prevent this ER stress, but *ORF3–* could not prevent ER stress, triggering premature programmed cell death, and leading to embryo-sac abortion (Chen et al., 2008; Yang et al., 2012). A tetratricopeptide repeat domain-containing protein encoded by *S7* was required for hybrid female sterility in the crosses between *aus* and *indica* or *japonica* (Yu et al., 2016). Two tightly linked genes, *HSA1a* and *HSA1b*, were responsible for hybrid female sterility in a cross between temperate *japonica* and *indica*. *HSA1a* encoded an unknown function protein with a highly conserved plant-specific domain, whereas *HSA1b*, encoded an uncharacterized protein with the nucleotide-binding domain. Heterozygous *HSA1a* with homozygous *HSA1b* allele from *indica* conferred hybrid sterility due to selective abortion of *HSA1a*-*i*^*s*^ gamete in the hybrid (Kubo et al., 2016a). Allelic suppression mediated by *Sc* contributed to hybrid male sterility between temperate *japonica* and *indica*. The *japonica* allele, *Sc-j*, encoded a DUF1618-domain protein; the *indica* allele, *Sc-i*, consisted of two or three tandem-duplicated *Sc-j* homolog. The selective abortion of *Sc-j* pollen leading to the transmission ratio distortion resulted from the expression of *Sc-j* suppressed by high expression of *Sc-i* in *Sc-j/Sc-i* hybrids (Shen et al., 2017). Hybrid pollen germination caused by reciprocal gene *DPL1/DPL2* was isolated from *japonica* and *aus* cross. DPL encoded a plant-specific small protein. The pollen from Nipponbare possessed *DPL1-N*^+^*/ DPL2-N*^−^ genotype, whereas pollen from Kasalath carried *DPL1-K*^−^*/ DPL2-K*^+^ genotype. Pollen harboring two nonfunctional alleles *DPL1-K*^−^ and *DPL2-N*^−^ failed to germinate and was not transmitted to the next generation (Mizuta et al., 2010). According to the above information, it is easy to find that the hybrid sterility of the different genetic materials was controlled by diverse genes or QTLs, and allelic interaction at each locus and non-allelic interaction at the different loci from two parents contribute to the intraspecific hybrid sterility. These genes or QTLs identified in the different populations will lay the foundation for elucidating the genetic and molecular mechanisms of hybrid sterility in Asian cultivated rice.

**Figure 2:**
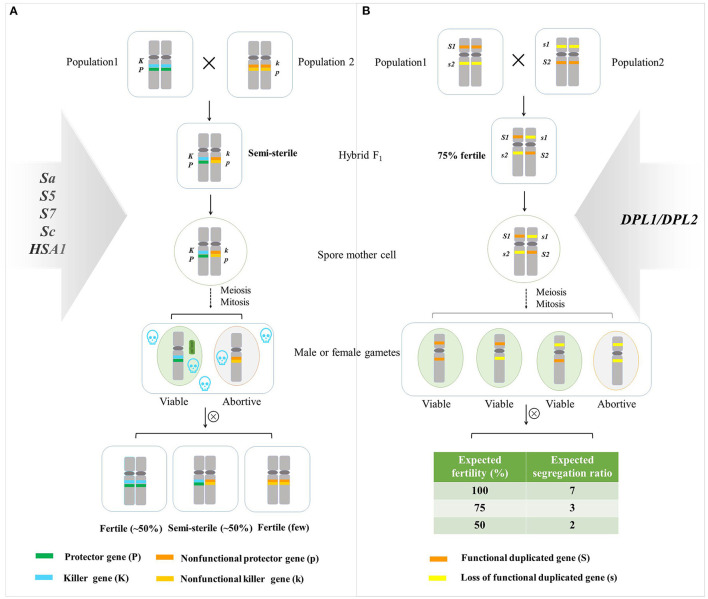
A genetic model of hybrid sterility. **(A)** Modified one-locus sporophytic–gametophytic allelic interaction model (Ikehashi and Araki, 1986). A hybrid sterility system consisted of at least two tightly linked genes: protector gene (P) and killer gene (K) in one population, nonfunctional protector gene (p), and nonfunctional killer gene (k) in another population. *PP* and *KK* constitute a functional killer system that triggers a sterility signal in the sporophytic stage, which selectively eliminates gametes without the *PP* gene, PP could protect the gametes in a gametophytic manner, leading to semi-sterility in the hybrid. Thus, the percentage of hybrid sterility plants in F_1_ hybrid accounted for 50%. The fertile plants and semi-sterile plants fit in with the ratio of 1:1 in the F_2_ population. *S5, S7, Sa, Sc*, and *HSA1* fitted in with this model (Chen et al., 2008; Long et al., 2008; Yang et al., 2012; Kubo et al., 2016a; Yu et al., 2016; Shen et al., 2017). **(B)** Duplicate gametic lethal model interpreting two independent loci involved in the hybrid sterility. When the gametes carrying *S1S1/s2s2* or *s1s1/S2S2* developed normally, whereas two recessive alleles at both loci, *s1s1/s2s2*, conferred deleterious interaction, and about 25% of gametes were sterile (Oka, 1957, 1974). The ratio of the fertile plants, partial fertile plants, and semi-sterile plants conforms to the theoretical ratio of 7:3:2 in the progeny. *DPL1/DPL2* followed this model (Mizuta et al., 2010).

Two genetic models were popular for explaining hybrid sterility. One model was the one-locus sporo-gametophytic interaction model (Kitamura, 1962), and another was a duplicate gametic lethal model interpreting two independent loci involved in the hybrid sterility (Oka, 1957, 1974). *S5, S7, Sa, Sc*, and *HSA1* fitted in with the one-locus sporo-gametophytic interaction model, and *DPL1/DPL2* followed the duplicate gametic lethal model (Figure 2; Long et al., 2008; Mizuta et al., 2010; Yang et al., 2012; Kubo et al., 2016a; Yu et al., 2016; Shen et al., 2017).

### Epistatic Interaction Between the Different Hybrid Sterility Loci

Hybrid sterility is a complex quantitative trait controlled by multiple loci, hence, it is conceived that gene–gene interaction is related to the sterility mechanism. Epistatic interaction between *S35* and *S24* regulated male sterility in rice intraspecific hybrid. *S24* locus in the heterozygous plants caused abortion of male gametes from the *japonica* allele, independent of the *S35* genotype, whereas male gametes from *japonica* alleles at the *S35* were aborted when the homozygous genomic fragment harboring the *S24* locus was introgressed into the heterozygous *S35* background plants (Kubo et al., 2000, 2008). Further study confirmed that a locus *INK* tightly linked with *S24*, not *S24*, activated *S35* to abort the male gametes from the *japonica* alleles at the *S35* heterozygous locus. Homozygous *EFS* allele from *indica* can activate the *S24* functioning in the pollen sterility, but the heterozygous *EFS* allele could eliminate the harmful effect of *S24* on the male gametes in the hybrid (Kubo et al., 2016b). Another sample was that heterozygous *HSA1a* locus from *indica* × *japonica* cross caused the female gamete abortion in the homozygous *HSA1b* allele from *indica*, Conversely, the female gamete from *japonica* at heterozygous *HSA1a* locus was normally developed with homozygous *HSA1b-j* allele (Kubo et al., 2016a). Four *S5*-interacting QTLs, *qSIG3.1, qSIG3.2, qSIG6.1*, and *qSIG12.1* were identified to interact with the *S5* locus by genetic mapping (Rao et al., 2021). These evidences suggested that a genetic network with multiple genetic factors played a pivotal role in the intraspecific hybrid sterility in rice. However, to date, the gene–gene interactions among hybrid sterility loci are not clear; one reason might be this interaction depends on the special genetic background, and no effective investigation method of gene–gene interaction between the different loci might be another reason. In the future, improvements in the quantitative genetics methods coupled with a representative number of rice varieties could be needed to clarify this issue. In addition, in terms of the genetic model, there remain some substantial holes in explaining the epistasis effect on hybrid sterility, thus more comprehensive model will be established in light of the complex genetic basis of hybrid sterility.

## Hybrid Sterility Loci and Divergence of Subgroups in Asian Cultivated Rice

### Different Hybrid Sterility Loci Were Involved in the Divergence Between Two Subgroups

**Figure 3:**
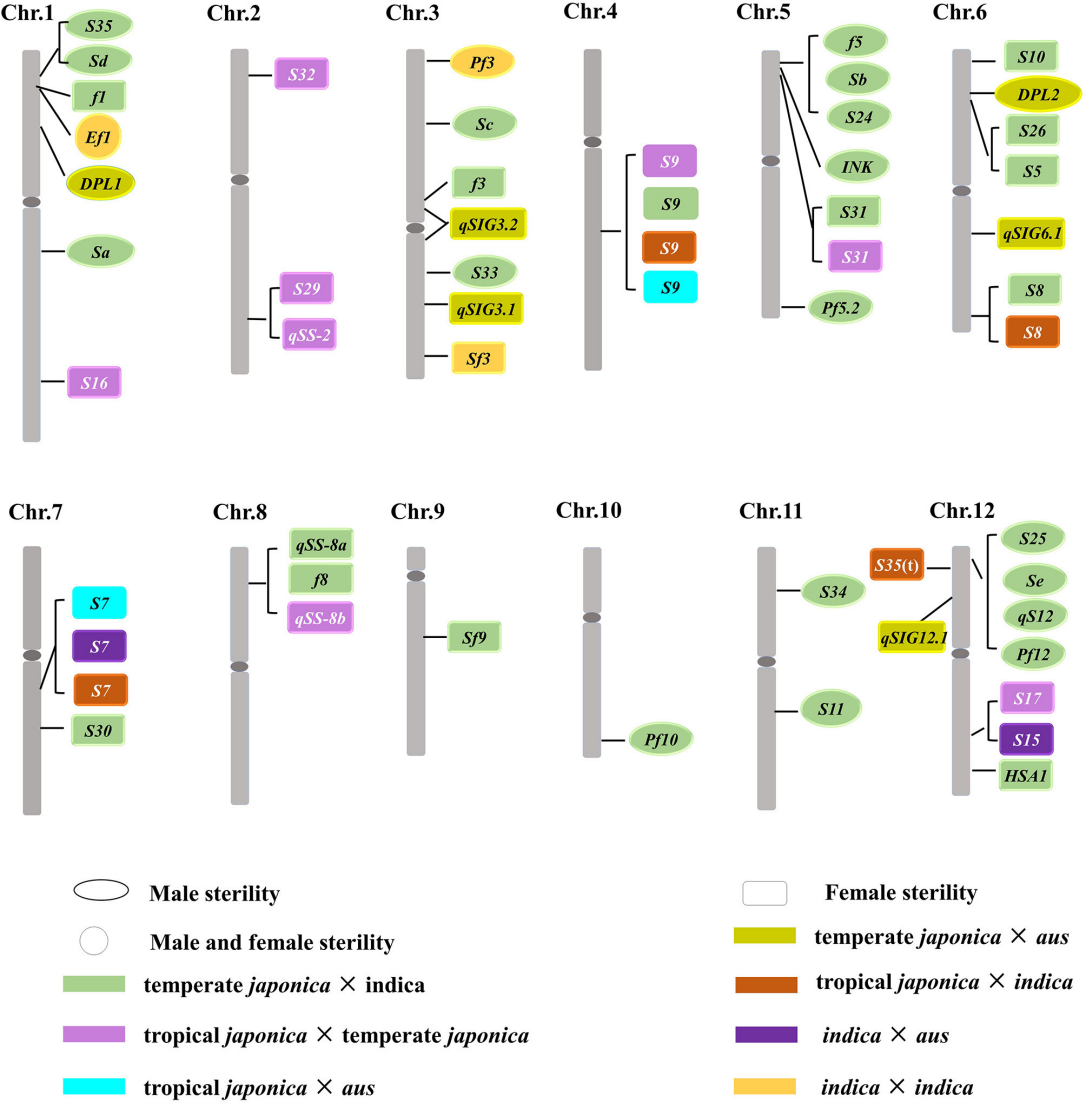
Loci for hybrid sterility in Asian cultivated rice.

Twenty-two hybrid sterility loci had been described between *indica* and temperate *japonica* cultivars (Figure 3; Supplementary Table 1), including *Sa, Sc, Sd, S11, S33, S34, S35, f5/Sb/S24, INK, Pf5.2, Pf10*, and *S25/Se/qS12/Pf12* for male gamete sterility (Sawamura and Sano, 1996; Kubo and Yoshimura, 2001; Yang et al., 2004; Li et al., 2006, 2008, 2017; Wang et al., 2006; Jing et al., 2007; Kubo et al., 2008; Long et al., 2008; Zhu et al., 2008; Zhang et al., 2011; Zhao et al., 2011; Shen et al., 2017) and *f1, f3, S5, S8, S9, S10, S30, S31, S5/S26, qSS-8a/f8, Sf9*, and *HSA1* for female gamete sterility (Sano et al., 1994; Wan et al., 1996; Wang et al., 1998, 2005; Kubo and Yoshimura, 2001; Zhu et al., 2005b; Singh et al., 2006; Zhao et al., 2006; Chen et al., 2008; Kubo et al., 2016a; Li et al., 2017). It is suggested that those loci are also involved in the divergence between temperate *japonica* and *indica* subgroups. Low-hybrid fertility was also reported among other subgroups in previous studies (Morinaga, 1968; Engle et al., 1969), and then, hybrid sterility loci were identified by genetic mapping in succession (Figure 3; Supplementary Table 1). *DPL1/DPL2* was responsible for hybrid pollen sterility (Mizuta et al., 2010), and *qSIG3.1, qSIG3.2, qSIG6.1*, as well as *qSIG12.1* gave rise to female gamete abortion in the crosses between temperate *japonica* × *aus* (Rao et al., 2021). These results indicated that *DPL1/DPL2, qSIG3.1, qSIG3.2, qSIG6.1*, and *qSIG12.1* were responsible for the divergence between temperate *japonica* and *aus* subgroups. *S7* and *S15* led to female gamete sterility in the hybrid between *indica* and *aus* (Wan et al., 1996; Yu et al., 2016), and it is suggested that both loci were involved in the reproductive isolation establishment between the two subgroups. Heterozygous loci at *S9, S16, S17, S29, S31, S32, qSS-2*, and *qSS-8b* resulted in gametes abortion in the hybrid between tropical *japonica* and temperate *japonica* (Wan and Ikehashi, 1995; Wan et al., 1998; Li et al., 2005, 2007; Wang et al., 2005; Zhu et al., 2005a; Zhao et al., 2007), which inferred that those loci acted in the genetic differentiation between tropical *japonica* and temperate *japonica*. In addition, *S7, S8, S*9, and *S35*(t) were responsible for the female sterility in the cross between tropical *japonica* and *indica* (Wan et al., 1993, 1996; Chen et al., 2012), it also indicated that those three loci promoted the differentiation between tropical *japonica* and *indica*. *S7* and *S9* controlled hybrid sterility and contributed to the divergence between tropical *japonica* and *aus* (Yanagihara et al., 1992; Wan et al., 1996). Taken together, the different combinations of distinct hybrid sterility loci could be the major driving force for Asian cultivated rice to differentiate into five subgroups, the type, and a number of loci involved in genetic differentiation varied with the subgroup types. The results supported the classification of five subgroups in Asian cultivated rice in terms of reproductive isolation.

### Allelic Differentiation at Hybrid Sterility Locus and Genetic Differentiation in Asian Cultivated Rice

In recent years, more and more evidences showed that some loci responsible for hybrid sterility were identified in several subgroups crosses simultaneously (Supplementary Table 1). *S7* contributed to the female sterility in the hybrid between tropical *japonica* or *indica* and *aus*, together with the tropical *japonica* and *indica* cross (Yanagihara et al., 1992; Wan et al., 1993; Yu et al., 2016), suggesting that the allelic differentiation at *S7* locus contributed to the divergence among tropical *japonica, indica*, and *aus* subgroups. *S8* locus conferring hybrid female sterility was reported in the different crosses between temperate *japonica* and *indica*, tropical *japonica* and *indica* (Wan et al., 1993; Singh et al., 2006), it indicated that *S8* had a potential to lead the genetic differentiation between *japonica* and *indica*. A locus *S9* for hybrid female sterility was found among the different subgroup crosses, including temperate *japonica* and tropical *japonica*, temperate *japonica* and *indica*, tropical *japonica* and *aus*, as well as tropical *japonica* and *indica* (Wan et al., 1996; Zhu et al., 2005a; Zhao et al., 2006), suggesting that allelic interaction at *S9* locus was involved in the divergence of the above subgroups. Heterozygous *S31* locus led to the hybrid female sterility in two kinds of populations, temperate *japonica* × *indica* and temperate *japonica* × tropical *japonica* (Li et al., 2005; Zhao et al., 2006), the same function had also been found in *qSS-8a/qSS-8b/f8* (Wang et al., 1998, 2005), it indicated that the genetic differentiation between temperate *japonica* and tropical *japonica* or *indica* could partially resulted from the interaction of the different haplotypes at two loci. Evidence indicated that *S15*/*S17* were mapped into the same region on chromosome 12, which might drove the divergence between *indica* and *aus*, temperate *japonica* and tropical *japonica*, respectively (Wan et al., 1996, 1998). Those six hot spots identified from different subgroup hybrids, with the same location region, as well as similar genetic behavior and phenotype, were probably orthologous genes and allelic to each other. It is suggested that these loci were conserved and contributed to hybrid sterility between different subgroups and allelic differentiation at hybrid sterility locus played important roles in triggering the divergence between distinct subgroups.

In a word, hybrid sterility is the major indication to distinguish the different subgroups in Asian cultivated rice at the subspecies level. A lot of genes or QTLs for male gamete abortion, female gamete abortion, and both male and female gamete abortions were identified in different populations. Some conserved loci governed the hybrid sterility among several subgroup crosses, whereas some loci were special for specific crosses; it suggested that multiple loci and allelic differentiation at hybrid sterility loci might be the important driving force of the divergence in Asian cultivated rice. However, the previous studies on hybrid sterility usually focused on the crosses between *japonica* and *indica*, with less progress on hybrid sterility between other subgroups. Future work should reinforce whole-genome-wide gene identification, and functional characterization of hybrid sterility loci in the hybrids between *aus* or *basmati* with other subgroups. It will help us to understand the genetic differentiation of subgroups and provide the guidelines for the heterosis strategy in rice breeding.

## The Origin, The Evolution of Hybrid Sterility Loci, and the Divergence of Asian Cultivated Rice

Hybrid sterility resulted in the female or/and male gametes abortion in the hybrid, which hindered gene exchange and had substantial effects on the divergence between the different populations and speciation. How the hybrid sterility loci originated and evolved, fixed in the different populations, and lead to the population divergence finally was one of the hot spots in evolutionary biology.

### *Sa, S5, Sc*, and *HSA1* Are Involved in the Differentiation of Temperate *Japonica* From *Indica*

For *Sa* locus, *SaF*^+^/*SaM*^+^ haplotype was a major form presented in ancient Oryza species, *SaF*^−^/*SaM*^+^ and *SaF*^−^/*SaM*^−^ haplotypes were generated by the sequential mutations in *SaF*^+^ and *SaM*^+^ in different accessions of *O. rufipogon*. And then, the increasing frequency of *SaF*^+^/*SaM*^+^ and *SaF*^−^/*SaM*^+^ fixed in *indica* populations, whereas the fixation of *SaM*^−^*SaF*^−^ in *japonica* rice could be resulted from random genetic drift, together with geographical isolation or ecological adaptation (Long et al., 2008). In the light of *SaF*^+^*, SaM*^+^, and *SaM* consisted of killer system, it is supposed that the hybrid fertility should be observed in the hybrid between *indica* and *O. rufipogon, indica* and *japonica*, or within *indica* subspecies, and then it was confirmed that *japonica* haplotype at the *Sa* locus contributed to the pollen abortion in the crosses between temperate *japonica* and *indica* or wild rice species (Long et al., 2008). Accordingly, the reproductive isolation mediated by the *Sa* locus contributed to the genetic differentiation between temperate *japonica* and *indica* subspecies.

By a reciprocal BLASTN search using three genes sequence, it was postulated that the *S5* complex could originated from Ospara3-5 by genome fragment duplication after Oryzeae tribe differentiation and produced a nascent loci *ORF3*+*ORF4*+*ORF5*+ with new introns and start codons. Most of the wild relatives in rice carried the functional *ORF3*+*ORF4*+*ORF5*+ allele. Mutations may occur in the partner gene by 11-bp deletion and killer gene by two SNP substitutions, leading to *ORF3*+*ORF4–ORF5*+ (the typical *indica* genotype), and *ORF3*+*ORF4*+*ORF5–* or *ORF3*+*ORF4*+*ORF5n*, respectively. And then, the protector *ORF3*+ mutated to *ORF3–* by 13bp deletion in the haplotypes of *ORF3*+*ORF4*+*ORF5–*, thus resulting in the typical *japonica* genotype of *ORF3–ORF4*+*ORF5–* in the pre-differentiated population. Subsequently, the increase of *indica* haplotype frequency was driven by natural selection, whereas the spread of *japonica* haplotype might be generated by the bottleneck during domestication. Once the frequencies of *ORF3*+*ORF4–ORF5*+ and *ORF3–ORF4*+*ORF5–* are fixed in the corresponding populations, *indica* and *japonica* subspecies would generate deleterious interactions upon hybridization, eventually resulting in reproductive isolation mediated by *S5* locus (Du et al., 2011; Mi et al., 2020). In previous reports, 72% accession of *O. rufipogon* carried the *ORF3*+*ORF4*+*ORF5*+ or neutral haplotype at the *S5* locus (Du et al., 2011; Mi et al., 2020) and this haplotype was compatible with the *japonica* or *indica* haplotypes. It suggested that it might be one of the reasons why hybrid sterility was universally observed in *indica* and *japonica* crosses, but almost never found in the crosses between *O. rufipogon* and *O. sativa*.

For the *HSA1* locus, the ancestral population of *O. rufipogon* possessed the fertile *HSA1a-j* and the sterile *HSA1b-i*^*s*^ alleles. Mutation in *HSA1a* of the *indica* ancestry and in *HSA1b* of the *japonica* ancestry occurred separately, which generated *indica* haplotype (*HSA1a*-*i*^*s*^*/HSA1b-i*^*s*^) and temperate *japonica* haplotype (*HSA1a-j/HSA1b-j*). The differences in adaptive and domesticated evolution at the *HSA1* locus accelerated the divergence between temperate *japonica* and *indica* subspecies (Kubo et al., 2016a). *Sc-j* and *Sc-i* haplotypes were found in the different *O. rufipogon* populations, suggesting that the origin of *Sc* occurred before the divergence between *indica* and *japonica* (Shen et al., 2017).

Therefore, hybrid sterility loci were diverged from the ancient haplotypes by a series of nucleotide variations and were further fixed in the *japonica* and *indica* subgroups, respectively, incompatible allelic interaction mediated by *Sa, S5, HSA1*, and *Sc*, activated the reproductive isolation system simultaneously, evenly restricting gene flow and resulting in the divergence of two subgroups.

### *DPL1/DPL2* Contributes to the Divergence Between Temperate *Japonica* and *Aus*

*DPL* originated from a small-scale genome duplication gene after the differentiation between *Oryza* and *Brachypodium*. Ancient *DPL* gene seemed to be functional by sequence analysis, but loss-of-function mutations of *DPL1* genes fixed in *indica* and its wild ancestor, *O. rufipogon*. Especially, all the examined accessions of *O. barthii* and *O. glaberrima* had a deletion in the *DPL1* exon, which suggested that the ancient *DPL1* allele experienced the process of many times genome deletion, nucleotide substitution, and deletion, and gradually established non-functional haplotype in the different population, and the *DPL2* gene defect was only found in the *japonica* cultivars (Mizuta et al., 2010). Based on the above data, hybrid pollen incompatibility governed by *DPL* had the potential to lead to reproductive isolation between *japonica* subspecies and *O. barthii* or *O. glaberrima*, but this phenomenon controlled by *DPL* has not been reported in interspecific hybridization population, there might be the function of *DPL* loci inhibited by an unknown factor during the stage of pollen germination. Furthermore, the origin and geographic distribution of *DPL* were investigated using 132 *O. sativa*, 296 accessions of *O. nivara*, and 388 accessions of *O. rufipogon. DPL1*^−^ and *DPL2*^−^ haplotypes of *O. sativa* separately emerged from an *O. nivara* population in India and *O. rufipogon* in South China, subsequently spread into other groups, especially *aus* and temperate *japonica*, respectively (Mizuta et al., 2010; Xu et al., 2021). Therefore, hybrid incompatibility established by *DPL* contributed to the genetic differentiation between *aus* and temperate *japonica* subspecies.

Taken together, the origin and evolution of *Sa* could be explained by the sequential divergence model, the mode of *S5* fitted well with the parallel-sequential divergence model, parallel divergence model could also be supported by the studies of *HSA1* and *DPL1/DPL2* (Ouyang and Zhang, 2018). Accordingly, these results supported the “multiple origins” hypothesis, in which the Asian cultivated rice has originated from the different wild relative groups. In the future, more and more cloned hybrid sterility loci would help understand the evolutional processes and history of Asian cultivated rice and find out the reasons for subspecies divergence governed by multiple hybrid sterility loci with complex mechanisms step by step. Moreover, the different haplotypes at each locus should be developed on the uniformed background as NILs to validate whether the haplotype combinations functioned in hybrid sterility or not, it will provide experimental evidence for the time and mechanism of reproductive isolation establishment.

## Strategies for Overcoming Hybrid Sterility in an Intraspecific Hybrid Breeding Program

**Figure 4:**
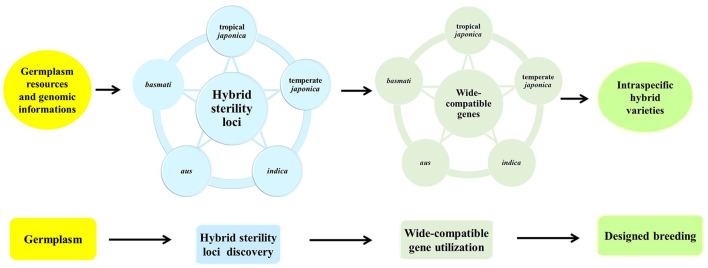
Strategies for developing intraspecific hybrid rice. The use of diverse rice accessions in five subgroups of Asian cultivated rice representing the abundant genetic diversity, together with plenty of genome information, the loci for intraspecific hybrid sterility among five subgroup crosses will be comprehensively and systematically explored, identified, and characterized by genome-wide genetic analysis, then natural neutral genes or wide-compatible genes will be created by CRISPR-Cas9 in light of special parental lines. Applying this information, breeders could design any intraspecific crosses using known wide-compatible genes, and will be able to breed fertile hybrid varieties with strong heterosis regardless of hybrid sterility.

Genetic diversity representing genetic differences between parental lines is fundamentally important to utilize heterosis in rice. Current hybrid rice varieties mainly utilize the heterosis from intra-subspecific crosses, mostly between *indica* parental lines. Hybrid yields have reached a bottleneck due to the limited genetic diversity in rice. Five subgroups in Asian cultivated rice possess abundant genetic diversity, which is the basis of strong heterosis. For example, *aus* subgroup showed drought-tolerant, early-maturing traits, and 11 of 143 heterotic loci were contributed by the introgression from *aus* varieties (Lin et al., 2020); *basmati* varieties possess excellent quality traits such as slender and long grain, fluffy and soft texture after cooking, and special aroma. Therefore, *basmati* is designated as the most high priced group (Civan et al., 2019). However, high genetic diversity between subgroups might reinforce sterility barriers to maintain the independent gene pools. How to break hybrid sterility and breed intraspecific hybrids with strong heterosis has always been explored by scientists for many years. So far, three strategies have been developed to overcome hybrid sterility and enable breeders to utilize the strong hybrid vigor of *O. sativa*. The first strategy was to transfer of neutral alleles of hybrid sterility loci into the target parents to raise wide-compatibility lines for hybrid breeding (Kitamura, 1962). Neutral allele introgression, including *S5-n, f5*, and *Sa-n*, have been proved to enhance the pollen or spikelet fertility in the hybrid (Mi et al., 2016, 2019; Xie et al., 2017a; Ma et al., 2020). The second strategy is to develop *indica*-compatible *japonica* lines by introgression and pyramid of multiple hybrid sterility loci from *indica* into the *japonica* genetic background by using molecular marker assistant backcrossing methods (Guo et al., 2016). The third strategy is to create an artificial wide compatible line by genome editing technology, such as CRISPR/cas9 (Xie et al., 2017a,b, 2019), which is a feasible, effective, and safe method. However, hybrid sterility is a complex trait controlled by multiple loci dependent on the genetic background. If wide compatible lines eliminating the effect of the hybrid sterile loci were generated by CRISPR/cas9, the conditions are required as follows: knowing all the loci for hybrid sterility between two parental lines, clearing killer genes in the different sterility systems. Accordingly, it is necessary to utilize abundant germplasm and genome information, develop mutual intraspecific populations with five subgroups, and explore all the hybrid sterility loci by genome-wide approach, as well as characterize the molecular mechanism of the sterility loci, so as to find out or artificially create neutral allele at each locus special for target parental lines. Based on the comprehensive and systematic data, breeders could effectively and accurately utilize the stronger intraspecific heterosis by genomic breeding (Figure 4).

## Perspectives

Five subgroups of Asian cultivated rice have provided a natural model to investigate the hybrid sterility. The genetic basis and gene identification of hybrid sterility in Asian cultivated rice have been described by many researchers, especially in *japonica* × *indica* crosses (Ouyang and Zhang, 2013, 2018; Li et al., 2020). But it is still difficult to utilize the intraspecific heterosis in rice breeding; this issue has many reasons: first, identifying only a few sterility loci is not sufficient to reveal the whole process of hybrid sterility. Second, complex variation in hybrid sterility in the hybrids differs from one cross to another, and most of the studies focus on hybrid sterility derived from one or few combinations on one genetic background, we know little about the type, number, and genetic mode of hybrid sterility loci in other subgroups, except for *japonica* and *indica*. Scattered information about sterile barriers leads to the low efficiency of intraspecific varieties improvement. There is a long way to understand the genetic basis and regulatory network of hybrid sterility.

### Comprehensive and Systematic Investigation of the Genetic Basis of Hybrid Sterility Based on Five Subgroups in Asian Cultivated Rice

To understand the genetic mechanism of hybrid sterility among the different subgroups comprehensively and systematically, the genetic population representing the typical five subgroups that crossed each other should be developed to elucidate the genetic basis of intraspecific hybrid sterility. Based on the amount of data in hybrid sterility special to parental lines, a blueprint for intraspecific hybrid sterility should be drawn in the distinct combinations derived from five subgroups, clearly marking the common or special features at gene types, gene numbers, conserved genes, special genes, major effect genes, minor effect genes, and gene–gene interaction, and so on. It would provide breeders with scientific and accurate guidelines to overcome the hybrid sterility special to target parental lines. Now, 20 varieties representing five subgroups were introgressed into a temperate *japonica* variety Dianjingyou 1 and an *indica* variety IR64 by multi-round backcrosses, respectively.

Moreover, *basmati* rice is considered a unique varietal group because of its aroma and superior grain quality (Ahuja et al., 1995; Siddiq et al., 2012; Civan et al., 2019) and it displays a different pattern of genetic diversity from other subgroups (Civan et al., 2019). Over the decades, less attention has been given to the hybrid sterility between *basmati* and other subgroups, though hybrid sterility was both observed in the hybrid between a *basmati* variety (Dom Sufid) and a *japonica* variety (Dianjingyou 1) or an *indica* variety (IR64) (data not shown). By now, none of the sterile loci was identified from the *basmati* variety, so, we should pay more attention to the hybrid sterility between the *basmati* group and other subgroups to utilize the genetic diversity and unique quality traits to breed intraspecific hybrid varieties.

### Molecular Mechanism and Signal Network of Hybrid Sterility Loci

To date, only six loci involved in intraspecific hybrid sterility were cloned (Chen et al., 2008; Long et al., 2008; Mizuta et al., 2010; Yang et al., 2012; Kubo et al., 2016a; Yu et al., 2016; Shen et al., 2017). We only see a few separating points, which cannot be connected to obtain a clear genetic and regulatory pathway. Thus, it is necessary to clone more genes, elucidate the allelic and non-allelic interaction, and characterize the molecular mechanism of hybrid sterility. Recent evidence indicated the DUF1618 protein not only played an important role in hybrid sterility, but also contributed to gametophyte development (Kubo et al., 2016a; Shen et al., 2017). However, its molecular function was still unclear. It will provide a rapid and easy way to overcome hybrid sterility if we can find out the “key nodes” or “switch” in the crosstalk network of hybrid sterility.

### Cloning and Characterization of Segregation Distortion Factors

Segregation distortion (SD) has substantial effects in population structure and fitness of the progenies, which would result in reproductive isolation and finally speciation. SD was observed widely in the cross populations, many loci for SD were identified by genetic analysis in rice (Figure 3; Kinoshita, 1991, 1993; Maekawa and Inukai, 1992; Rha et al., 1995; Xu et al., 1997; Harushima et al., 2001; Matsushita et al., 2003; Reflinur et al., 2014; Li et al., 2019; Xia and Ouyang, 2020). However, only one locus *DPL1/DPL2* has been cloned and characterized (Mizuta et al., 2010), genetic and molecular mechanism of other loci remains to be unclear. Understanding the molecular events controlling SD will shed light on the reproductive isolation and speciation, as well as provide a potential approach to keep genetic diversity in populations. The above-mentioned studies will broaden our knowledge about hybrid sterility and divergence of Asian cultivated rice, and overcome the reproductive isolation so as to serve for rice intraspecific breeding.

## Conclusion

In this article, we reviewed the comprehensive studies on the genetic differentiation and the origin of Asian cultivated rice, the comparative analysis of hybrid sterility loci among five subgroups, as well as an illustration of the relationships between hybrid sterility loci and the differentiation of subgroups in *O. sativa*. Clearly, Asian cultivated rice possesses abundant genetic diversity, which reflects the striking differences in traits, physiology, genome structure, geographic distribution, and ecological adaptation, accompanied by human civilization. Muti-dimensional discrepancies, including hybrid sterility, drive the genetic differentiation in rice. Conserved and special sterile loci contribute to the Asian cultivated rice divergence. Based on the genetic diversity of subgroups and information on the rice genome, it will facilitate gene flow by utilizing wide compatible genes for future rice breeding. Identifying loci conferring hybrid sterility between subgroups by genome-wide analysis, and elucidating the molecular mechanism and signal network will be easy to overcome the hybrid sterility by precise gene modification of key “killer genes” or “switch gene” in the sterility system.

## Author Contributions

DT proposed the concept. YZ conceived and wrote the paper. JW, QP, YY, YL, JZ, JL, XD, and MW edited the manuscript. All the authors reviewed and approved the final manuscript.

## Funding

This research was supported by the National Natural Science Foundation of China (Grant Nos. 31991221, 31660380, 31201196, and 32160489), Yunnan Fundamental Research Projects (202001AS070003, 202101AS070036, 202101AT070193, and 530000210000000013809), the Yunnan Provincial Government (YNWR-QNBJ-2018-359), and the Yunnan Seed Industrialization Laboratory Program.

## Conflict of Interest

The authors declare that the research was conducted in the absence of any commercial or financial relationships that could be construed as a potential conflict of interest.

## Publisher's Note

All claims expressed in this article are solely those of the authors and do not necessarily represent those of their affiliated organizations, or those of the publisher, the editors and the reviewers. Any product that may be evaluated in this article, or claim that may be made by its manufacturer, is not guaranteed or endorsed by the publisher.
