# Supplementary material for: Understanding the Nature of Hybrid Sterility and Divergence of Asian Cultivated Rice
Source: Front Plant Sci. 2022 Jun 27;13:908342. doi: 10.3389/fpls.2022.908342 (PMC9272003; doi:10.3389/fpls.2022.908342)
Supplement: Supplementary file 1 [file Table_1.DOCX]

| Table S1. Loci for hybrid sterility in Asian cultivated rice | | | | | |
| --- | --- | --- | --- | --- | --- |
| Loci | Chr. | Genomic location | Aborting gamete | cross | Reference |
| *S5* | 6 | RG213-Est2 | ♀ | Temperate *japonica*×*indica* | Chen et al*.,* 2008,  Yang et al*.,* 2012 |
| *S7* | 7 | Rc-Est-9 | ♀ | Tropical *japonica*× *aus,*  *indica*×*aus,*  Tropical *japonica× indica* | Yanagihara et al*.,* 1992,  Wan *et al*.,1993,  Wan et al.,1998,  Yu *et al.,* 2016 |
| *S8* | 6 | Cat-1-Pox-5 | ♀ | Tropical *japonica*×*indica,*  Temperate *japonica× tropical japonica,* Temperate *japonica×indica* | Wan et al*.,* 1993,  Wan et al.,1998,  Singh et al., 2006 |
| *S9* | 4 | RM185-RM3742 | ♀ | Tropical *japonica×indica,*  Tropical *japonica×aus,*  Temperate *japonica×* tropical *japonica,* Temperate *japonica×indica* | Wan et al*.,* 1996,  Zhu et al.,2005a  Zhao et al.,2006 |
| *S10* | 6 | linked with *Wx* gene | ♀ | Temperate *japonica* ×*indica*  *indica* ×*Weedy rice* | Sano et al*.,* 1994,  Zhu et al.,2005b |
| *S11*(t) | 11 | linked with *la* gene | ♂ | Temperate *japonica* ×*indica,* | Sawamura et al., 1996 |
| *S15* | 12 | Pox2-Sdh1 | ♀ | *aus*×*indica*, | Wan et al., 1996 |
| *S16* | 1 | hsp3-ch16 | ♀ | Temperate *japonica*× tropical *japonica* | Wan et al., 1995 |
| *S17* | 12 | C2-C751 | ♀ | Temperate *japonica*× tropical *japonica* | Wan et al., 1998 |
| *S24*(t) | 5 | R830-R3166 | ♂ | Temperate *japonica* ×*indica* | Kubo et al., 2000,  Zhao et al., 2011 |
| *S25*(t) | 12 | G24-G189 | ♂ | Temperate *japonica* ×*indica* | Kubo et al., 2001 |
| *S26*(t) | 6 | L688-R2171 | ♀ | Temperate *japonica* ×*indica* | Kubo et al., 2001 |
| *S29*(t) | 2 | RM8255-RM425 | ♀ | Temperate *japonica* ×tropical *japonica* | Zhu et al*.* 2005a |
| *S30*(t) | 7 | RM11-RM 432 | ♀ | *indica* ×*Weedy rice* | Zhu et al*.* 2005b |
| *S31*(t) | 5 | RM5586-RM13 | ♀ | Temperate *japonica*×*indica*,  Temperate *japonica*× tropical *japonica* | Li et al., 2005,  Zhao et al. 2006,  Li et al.,2007  Zhao et al*.* 2007 |
| *S32*(t) | 2 | RM236-RM211 | ♀ | Temperate *japonica*× tropical *japonica* | Li et al., 2005,  Li et al.,2007 |
| *S33*(t) | 3 | RM15621-RM15627 | ♂ | Temperate *japonica*×*Weedy rice* | Jing et al., 2007 |
| *S34*(t) | 11 | RM167-RM552 | ♂ | Temperate *japonica*×*Weedy rice* | Jing et al., 2007 |
| *S35*(t) | 12 | RM19-RM6269 | ♀ | Tropical *japonica*×*indica* | Chen et al*.,* 2012 |
| *S35* | 1 | RM6324-RM8105 | ♂ | Temperate *japonica* ×*indica* | Kubo et al*.,* 2008 |
| *Sa* | 1 | CDO568(6.4cM) | ♂ | Temperate *japonica* ×*indica* | Long et al*.,* 2008 |
| *Sb* | 5 | PSM8-PSM202 | ♂ | Temperate *japonica* ×*indica* | Li et al*.,* 2006 |
| *Sc* | 3 | RG227STS-RM218 | ♂ | Temperate *japonica* ×*indica* | Yang et al., 2004,  Shen et al*.,* 2017 |
| *Sd* | 1 | PSM13-RM283 | ♂ | Temperate *japonica* ×*indica* | Zhang et al*.,* 1994  Li et al., 2008 |
| *Se* | 12 | PSM180-PSM182 | ♂ | Temperate *japonica* ×*indica* | Zhang et al., 1994  Zhu et al*.* 2008 |
| *Sf* |  |  | ♂ | Temperate *japonica* ×*indica* | Zhang et al*.* 1994 |
| *hsa1* | 12 | *SP-5229-SP-5232* | ♀ | Temperate *japonica* ×*indica* | Kubo et al. 2016 |
| *qS12* | 12 | MS062-MS102 | ♂ | Temperate *japonica* ×*indica* | Zhang et al. 2011 |
| *DPL1* | 1 | S11214 | ♂ | Temperate *japonica*× *aus* | Mizuta et al. 2010 |
| *DPL2* | 6 | S1520 | ♂ | Temperate *japonica*× *aus* | Mizuta et al. 2010 |
| *f1* | 1 | RG532-R3129 | ♀ | (Temperate *japonica* ×*aus*) ×*indica* | Wang et al., 1998 |
| *f3* | 3 | RG393-C603 | ♀ | (Temperate *japonica* ×*aus*) ×*indica* | Wang et al., 1998 |
| *f5* | 5 | R830-R3166 | ♂ | (Temperate *japonica* ×*aus*) ×*indica* | Wang et al., 1998, Wang et al., 2006 |
| *f8* | 8 | C1121A-RG333 | ♀ | (Temperate *japonica* ×*aus*) ×*indica* | Wang et al., 1998 |
| *Pf3* | 3 | Chr03_bin175 | ♂ | *indica*×*indica* | Li et al., 2017 |
| *Pf5.2* | 5 | Chr05_bin213 | ♂ | Temperate *japonica* ×*indica* | Li et al., 2017 |
| *Pf10* | 10 | Chr10_bin145 | ♂ | Temperate *japonica* ×*indica* | Li et al., 2017 |
| *Pf12* | 12 | Chr12_bin15 | ♂ | Temperate *japonica* ×*indica* | Li et al., 2017 |
| *Ef1* | 1 | Chr01_bin33 | ♂/♀ | *indica*×*indica* | Li et al., 2017 |
| *Sf3* | 3 | Chr03_bin175 | ♀ | *indica*×*indica* | Li et al., 2017 |
| *Sf9* | 9 | Chr09_bin34 | ♀ | Temperate *japonica* ×*indica* | Li et al., 2017 |
| *qSS-2* | 2 | RM263 | ♀ | Temperate *japonica*× tropical *japonica* | Wang et al.,2005 |
| *qSS-8a* | 8 | XNpb278 | ♀ | Temperate *japonica* ×*indica* | Wang et al.,2005 |
| *qSS-8b* | 8 | RM6863 | ♀ | Temperate *japonica*× tropical *japonica* | Wang et al.,2005 |
| *qSIG3.1* | 3 | C0326556-C0328430 | ♀ | Temperate *japonica* × *aus* | Rao et al., 2021 |
| *qSIG3.2* | 3 | C0317639-C0323537 | ♀ | Temperate *japonica* × *aus* | Rao et al., 2021 |
| *qSIG6.1* | 6 | C0625165-C0626545 | ♀ | Temperate *japonica* × *aus* | Rao et al., 2021 |
| *qSIG12.1* | 12 | C124702-C125855 | ♀ | Temperate *japonica* × *aus* | Rao et al., 2021 |
